# Long-range proton-coupled electron transfer in biological energy conversion: towards mechanistic understanding of respiratory complex I

**DOI:** 10.1098/rsif.2017.0916

**Published:** 2018-04-11

**Authors:** Ville R. I. Kaila

**Affiliations:** Department of Chemistry, Technische Universität München, Lichtenbergstr. 4, Garching, Germany

**Keywords:** NADH:ubiquinone oxidoreductase, PCET, proton transfer, molecular simulations, bioenergetics

## Abstract

Biological energy conversion is driven by efficient enzymes that capture, store and transfer protons and electrons across large distances. Recent advances in structural biology have provided atomic-scale blueprints of these types of remarkable molecular machinery, which together with biochemical, biophysical and computational experiments allow us to derive detailed energy transduction mechanisms for the first time. Here, I present one of the most intricate and least understood types of biological energy conversion machinery, the respiratory complex I, and how its redox-driven proton-pump catalyses charge transfer across approximately 300 Å distances. After discussing the functional elements of complex I, a putative mechanistic model for its *action-at-a-distance* effect is presented, and functional parallels are drawn to other redox- and light-driven ion pumps.

## 1. Introduction

Energy conversion in nature is driven by efficient enzymes that catalyse elementary transfers of protons and electrons, or proton-coupled electron transfer (PCET) reactions [1–4]. Two main PCET pathways establish the bioenergetic basis of all life forms: the light-driven PCET of photosynthesis and the chemically driven PCET of respiratory chains. The photosynthetic PCET reactions catalyse splitting and oxidation of water into protons and electrons in green plants and cyanobacteria [5]. The electrons released from the water oxidation process are further employed to synthesize complex organic molecules from simple inorganic ones, such as CO_2_. Molecular oxygen (O_2_), which is released as a ‘waste’ product from photosynthesis, powers respiratory chains of mitochondria and aerobic bacteria, where the electrons extracted from foodstuffs, catalyse proton transfer reactions across biological membranes [6–8]. These charge transport processes establish an electrochemical proton gradient or proton motive force (*pmf*) across the biological membrane that is used to thermodynamically drive endergonic processes in the cell such as synthesis of adenosine triphosphate (ATP) and active transport [9,10].

**Figure 1.**
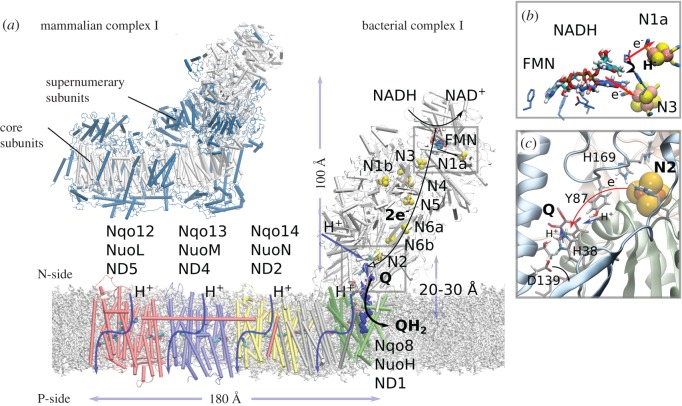
Structure and function of complex I. (*a*) The hydrophilic domain of complex I catalyses hydride transfer from NADH to FMN that triggers a stepwise electron transfer along a chain of eight FeS centres to quinone (Q), located approximately 20–30 Å above the membrane plane. The quinone-reduction process is coupled to proton pumping across the antiporter-like membrane subunits Nqo14, Nqo13, Nqo12 and Nqo8. The structural model was built based on complex I from *Thermus thermophilus* (PDB: 4HEA). *Inset*: the bovine complex I (PDB ID: 5LC5) showing the supernumerary subunits (in blue) that surround the core complex (in white). (*b*) Close-up of the NADH/FMN site (PDB ID: 3IAM). (*c*) Close-up of the N2/Q-reduction site, with Q in its stacking conformation with His-38 (see text). The figure was prepared with VMD [15].

Complex I or NADH:ubiquinone oxidoreductase functions as the initial electron entry point in aerobic respiratory chains [11–14]. It is by far the largest and most intricate member of the bacterial and mitochondrial respiratory chains, and it thus provides an excellent system to understand fundamental mechanistic principles of long-range PCET reactions. This 0.5–1 MDa L-shaped enzyme (figure 1) comprises 14 core subunits that are highly conserved from bacteria to eukaryotes, and up to 32 additional supernumerary subunits that provide an outer shell around the core subunits in eukaryotes (figure 1*a*) [16–19]. The supernumerary subunits are involved in modulating the activity of the enzyme (see below, [20,21]).

Complex I receives electrons from reduced nicotinamide adenine dinucleotide (NADH) and transfers them to quinone (Q) in its hydrophilic domain, which is coupled to the pumping of four protons across the membrane domain [22–26]; but cf. also [27]. The resulting quinol, QH_2_, diffuses further to the respiratory complex III (cytochrome *bc*_1_) from where the electrons continue their journey to cytochrome *c* oxidase (C*c*O complex IV) [8]. C*c*O reduces dioxygen to water, and provides a thermodynamic driving force for the complete respiratory chain [2,7,28]. This PCET chain spans a redox drop of approximately 1.1 V from NADH (−320 mV) to O_2_ (+810 mV) that is employed for the transfer of protons across the membrane against a *pmf* of approximately 200 mV that is established over the approximately 30 Å thick biological membrane.

Complex I is the only known enzyme that catalyses a long-range PCET process that extends a remarkable distance of approximately 300 Å between the tip of its electron entry site for NADH and the terminal proton-pumping subunit (figure 1, Nqo12/NuoL/ND5; subunit numbering refers here to *Thermus thermophilus*, *Escherichia coli* and *Bos taurus* enzymes; the *E. coli* notation is used here in the general mechanistic discussion). Complex I separates the elementary charge transfer processes to local electron and proton transfer reactions that are employed for pumping protons across the complete membrane domain. The Q-reductase activity at the ‘lower edge’ of the hydrophilic domain of complex I and the proton-pumping activity in its membrane domain are fully reversible and coupled. Complex I can therefore also catalyse QH_2_ oxidation and reverse electron transfer by employing an external pH gradient that reduces NAD^+^ into NADH [29,30]. Such operation modes are particularly relevant under certain physiological conditions, such as hypoxia or ischaemia [31], where C*c*O cannot thermodynamically drive the electron transport chain. Owing to this reversibility, mutation of residues involved in proton pumping in distant membrane-bound subunits (NuoL/Nqo12/ND5) also leads to the inhibition of the Q-reduction activity [32,33]. Although this is consistent with the principle of microscopic reversibility, the molecular basis of how such a long-range, *action-at-a-distance* coupling is achieved remains unanswered.

In this work, the structure, dynamics and energetics of complex I is discussed based on data from recent biochemical, biophysical and computational experiments. A mechanistic model that could explain central parts of the long-range proton–electron transfer process is also presented.

## 2. Long-range electron transfer

Electron transport is a key process in biological energy transduction. Owing to their small mass, electrons undergo quantum mechanical tunnelling, up to approximately 14 Å distances in biological systems [34,35], without the need of a physical conduction pathway (but cf. [36,37]), such as a hydrogen-bonded network that is a prerequisite for transporting protons [38] (see below). Owing to the tunnelling process, the surrounding nuclei do not have time for reorganization, and the electron transfer usually takes place non-adiabatically. Based on Fermi's golden rule expression for vertical excitation processes, Marcus, Levich and Dogonadze showed [39–42] that the rate for the non-adiabatic electron transfer, *k*_eT_, depends on three parameters: the electronic coupling, |*H*_ab_|^2^, which is a measure of the “interaction” strength between the donor and acceptor groups, and is defined as the splitting between non-adiabatic and adiabatic potential energy curves, the reorganization energy, *λ*, which measures the energy required to transfer the electron to oxidized nuclear geometry of the acceptor site, and the free energy of the transfer process, Δ*G*:
2.1
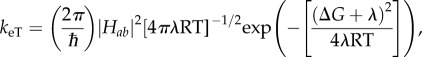
where *ħ* is Planck's constant divided by 2*π*, *R* is the gas constant and *T* is the temperature. The electronic coupling, *H_ab_*, decays exponentially with the distance between the electron donor and acceptor, *r*, exp(−*γr*), where *γ* is a constant that depends on the medium [36,37].

Dutton and co-workers [34,35] employed a quantized version of the Marcus equation (2.1) [43,44] in combination with empirical parameters to obtain
2.2
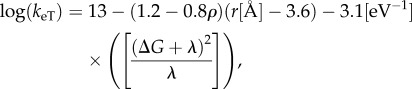
where *r* is the *edge-to-edge* distance between the donor and acceptors sites, and *ρ* is the protein packing density, which in many proteins is approximately 0.76 [35]. Equation (2.2) thus provides a practical way to estimate electron transfer rates based on structural (*r, ρ*) and thermodynamic (*G, λ*) parameters in biological systems, such as the electron transfer chain in complex I (see below).

Complex I employs its hydrophilic domain for electron transfer between NADH and the Q site. The electron transfer domain comprises an NADH/flavin mononucleotide (FMN) site and a chain of 8–9 iron–sulfur (FeS) centres that have evolved from NiFe and FeFe hydrogenases [45–47]. The terminal Q-reduction site is located approximately 20–30 Å ‘above’ the membrane plane [48,49], whereas the FeS chain extends up to approximately 100 Å from the membrane domain (figure 1), and does thus reside beyond the electric Stern layer [50], where ions (protons) are expected to adsorb, and above which the potential drops exponentially towards the diffusive layer, where ions are evenly distributed. Variations in the *pmf* across the membrane are, therefore, not expected to strongly modulate the intrinsic rate of either the forward or reverse electron transfer process. The spatial separation of the electron transfer domain from the membrane plane could thus secure a steady rate of electron transfer, with a possible physiological relevance.

Structural data [51,52] show that the NADH/FMN site is located in an unusual Rossmann fold that comprises four β-sheets instead of the typical six β-sheet structures. The NADH forms π-stacking interactions at an approximately 3.5 Å distance with the FMN isoalloxazine ring, which bridges the hydrogen to an approximately 2.6 Å distance from the FMN group (figure 1*b*). The surrounding residues are likely to facilitate the hydride transfer process between NADH and FMN, e.g. a conserved Glu-97 (all amino acids refer here to the *T. thermophilus* numbering if not otherwise stated; Glu-95 in *E. coli*) has been suggested to tune the redox potential of FMN by approximately 40 mV [53], and some mutations close to the FMN site have been linked to disease. For example, the Leigh syndrome mutation Y180C/C182G [54] is expected to break the hydrogen bond between Tyr-180 and Glu-97. Interestingly, half of all human mitochondrial disorders have been associated with mutations in complex I [11,31,55]. These mutations are located at the FMN site, Q-binding pocket, or at the first membrane-bound subunit, NuoH (Nqo8/ND1) [56–58], which couples the electron and proton transfer modules of complex I together.

The electron transport process in complex I is initiated by transfer of two electrons and one proton, i.e. effectively a hydride ion (H^−^), from NADH to FMN at a rate of approximately greater than 15 000 s^−1^ [59] (Δ*G*^‡^ < 12 kcal mol^−1^). No flavosemiquinone (FMNH^•^) signals have so far been detected, suggesting that the process takes place concertedly. The formation of FMNH^−^ is followed by transfer of the two electrons into the approximately 100 Å long FeS chain that comprises both binuclear (2Fe-2S) and tetranuclear (4Fe-4S) FeS clusters. The FeS clusters in complex I are one-electron acceptors that shuttle between their Fe^III^Fe^III^ / Fe^II^Fe^III^ and 2Fe^II^2Fe^III^ / 3Fe^II^Fe^III^ forms [60], and the electrons, therefore, bifurcate after FMN to N1a and N3 (figure 1*b*), as possible initial acceptor sites [61]. The NADH domain of complex I thus functions as a *two-to-one* electron converter. The subsequent oxidation of FMNH^−^ might reduce the affinity of the formed NAD^+^ due to weakening of electrostatic interaction between the two species. The role of the nearby N1a FeS cluster still remains unclear, as this cluster is difficult to reduce in some species [62]. Recent experiments [63], however, suggest that the reduction of N1a increases the affinity for NAD^+^, which might be important for preventing the electrons from leaking to the solvent that could, in turn, lead to formation of reactive oxygen species (ROS). Such gating could be achieved in part by an order of magnitude stronger affinity of NADH (*K*_m_^NADH^ ≈ 1 µM) relative to NAD^+^ (*K*_m_^NAD+^ ≈ 1 mM), and a slow (millisecond) dissociation of NAD^+^ from the FMN site [61] that might kinetically favour the electron transfer from FMN/N3 towards N2 rather than to the bulk.

Most FeS centres in complex I are nearly equipotential, close to −320 mV relative to the NHE [60,61,64–66], and thus nearly isoenergetic with the NADH/NAD^+^ redox couple. The terminal N2 FeS centre is an exception, with an *E*_m_ of approximately −150 … −200 mV depending on the species [62,67], and it functions thus as a sink for the electron transport chain. The higher redox potential of N2 relative to the other FeS centres is established by replacing backbone hydrogen bonding around the FeS core with positively charged arginine residues (figure 1*c*; [68]). Moreover, the nearby His-169 has been shown to undergo protonation changes upon reduction of N2 [69]. The N2 centre has also an interesting tandem cysteine motif (Cys-45 and Cys-46) [51], which affects its electronic structure by energetically splitting different spin states [68]. Despite its importance in the terminal electron transfer step, recent experiments [70] suggest that the reduction of N2 is not linked with the major energy conversion steps in complex I (see below).

The FeS clusters are located at an approximately 9–14 Å *edge-to-edge* distance from each other except N7, which is approximately 22 Å off-pathway from N3 and does not participate in the electron transfer process (figure 1; [61,71]). A bottleneck in the electron transfer process is established by the N5 centre, which is separated by approximately 14 Å from N6a. In N5, one of the cysteine residues is replaced by a histidine ligand, which is likely to increase the redox potential of the centre. Interestingly, experiments [72] suggest that reduction of N2 slows down the N5 → N6a electron transfer rate, which could provide a feedback regulation mechanism, e.g. under reverse electron transfer conditions.

Verkhovskaya *et al*. [61] measured, based on EPR freeze-quenched experiments that the electron transfer between NADH and the N2 centre takes place on approximately 90 µs timescales. Interestingly, electron transfer rates estimated based on equation (2.2) [35,61,73] using standard reorganization energies (*λ* = 0.7 eV) and packing densities (*ρ* = 0.76), structural data (*edge-to-edge* distances from PDB ID:2FUG), and measured *E*_m_ values suggest that the overall electron transfer rate between FMN and Q is found to be in the 100 µs domain, which is consistent with the experimental rates [61,72]. This suggests that the process might involve only quantum mechanical tunnelling, instead of, e.g. coupled conformational changes, which would be expected to slow down the process [61]. However, using redox potentials and pairwise interactions of the FeS centres obtained from electrostatic calculations [65,66], the predicted rate is in the milliseconds timescale. This is probably too slow to support the overall millisecond turnover rate of complex I [12], because the electron transfer is not expected to be rate-limiting in complex I. This effect might, however, arise in part from challenges to predict *E*_m_ values for FeS centres with an accuracy higher than 0.2 eV [68], which can affect the predicted rates by an order of magnitude. Moreover, semi-empirical calculations [74,75] suggest that the computed electronic coupling between N5 → N6a leads to an electron transfer rate of approximately 10 s^−1^, which is indeed too slow to support the experimental transfer rates. It was, therefore, suggested that structurally unresolved water molecules could help to increase electronic couplings between these centres. Microsecond-timescale molecular dynamics (MD) simulations [76,77] indeed show that water molecules accumulate between some of the FeS centres. However, explicit determination of reorganization energies, free energies, as well as first-principle electronic couplings for the pairwise electron transfer steps are currently required to determine the exact mechanisms and regulation of the electron transfer process. To this end, Martin & Matyshov [78] recently found that the reorganization energies for the N4, N5 and N6a centres are in the range of 0.6–1.1 eV.

## 3. Quinone chemistry and dynamics

There are to date no refined X-ray or cryo-EM structures of Q bound to complex I, but biochemical experiments show that Tyr-87 and His-38 are important for the Q-reductase activity [79,80]. Moreover, recent computational work [68] identified that His-38 can form both stacking and hydrogen-bonded interactions with Q (figure 1*c*), with the former conformation being thermodynamically slightly preferred when Q is oxidized. In several Q-oxidoreductases such as complex III [81] and photosystem II [82], the Q-headgroup binds within the membrane plane of the enzymes. By contrast, the Q headgroup in complex I binds approximately 20–30 Å above the membrane plane [48]. Another difference is also that both complex III and photosystem II operate with two quinones, whereas complex I is likely to bind only one ubiquinone molecule [83], which, however, might shuttle between two sites (see below).

**Figure 2.**
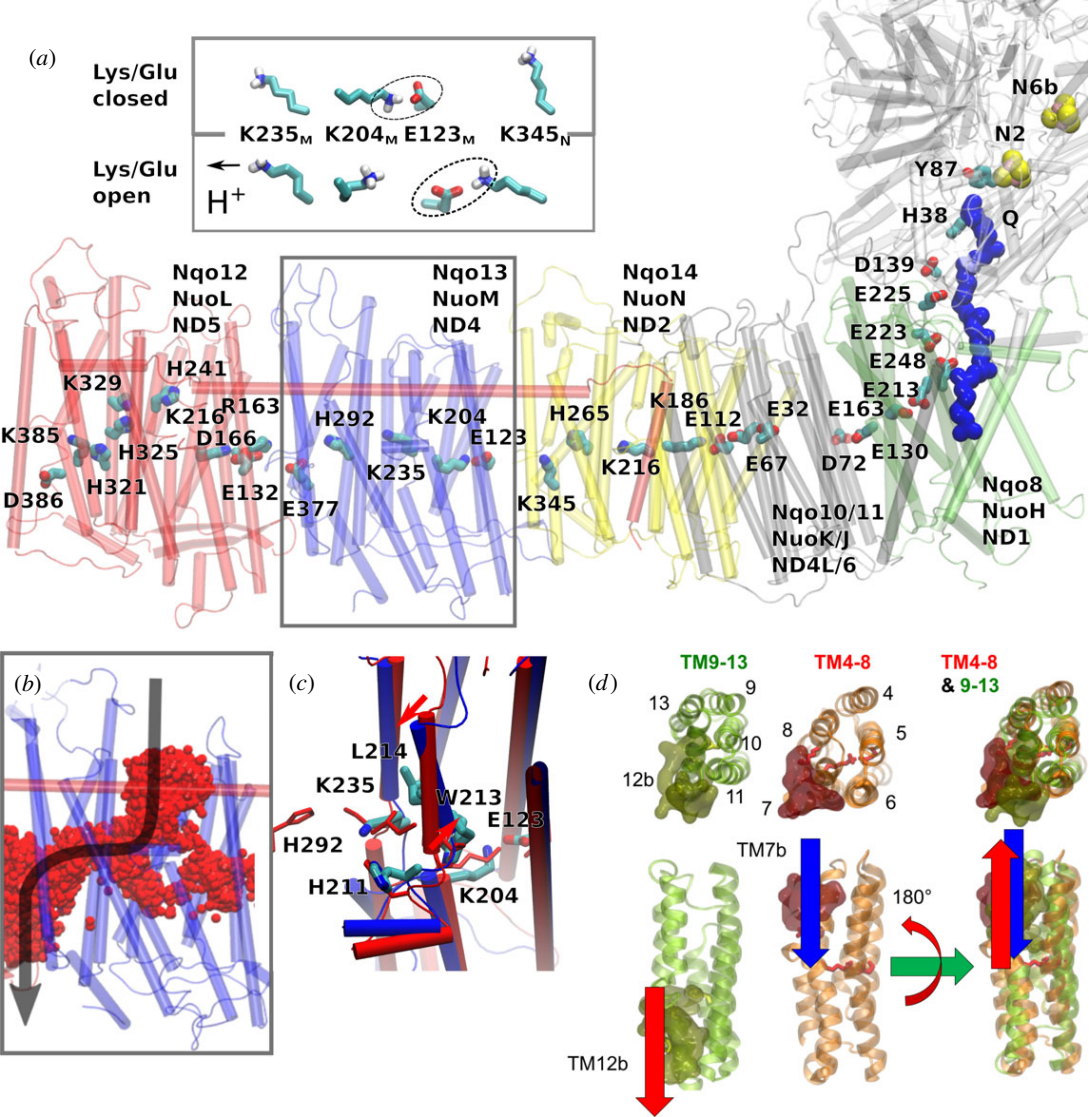
Structure of the proton-pumping membrane domain in complex I. (*a*) The membrane domain of complex I showing conserved buried charged/hydrophilic residues. NuoL (red); NuoM (blue); NuoN (yellow); NuoH (green); Q (blue van der Waals representation). *Inset*: The conformation of the Lys/Glu ion pair (here Lys-204_M_/Glu-123_M_) can modulate the p*K*_a_ of the middle Lys (Lys-235_M_). (*b*) Water molecules (in red) establish protonic connectivity in all antiporter-like subunits. The figure shows time-averaged occupancies of water molecules, i.e. water molecules that visit the channel area during the simulation time, in the NuoM subunit based on microsecond MD simulations. (*c*) Snapshot of structures obtained from MD simulations of open (in blue) and closed (in red) proton channels from the N-side in Nqo13 (NuoM/ND4), showing conformational changes in the broken helix element. (*d*) The structural symmetry of the antiporter-like subunits with an N-side input channel near broken helix TM7b and output channel near broken helix TM12b. The two five-helical bundles (TM4–8 and TM9-13) are related by rotation and translation symmetry, which is different from the rotation–inversion symmetry found in typical transporters.

The Q-tunnel has a restriction site in the NuoH subunit, where the isoprenoid tail bends in a approximately 90° angle towards the membrane plane ([49,84], figure 2). The Q-isoprenoid tail moves along this tunnel that is formed between the NuoH/NuoCD/NuoB subunits. Both non-polar and charged residues surround the tunnel edge [49,84], and the exit site forms between three helices in NuoH, approximately 10 Å below the membrane plane (figure 2). It was initially suggested that the Q-tunnel is tightly sealed from water molecules, but recent MD simulations suggest that the Q-headgroup and parts of the E-channel in NuoH (see below) become hydrated [76,85]. These observations imply that there could be accessible pathways to re-protonate Tyr-87 and/or His-38 after Q reduction (see below), but also that the Q-dynamics could link with proton uptake in the NuoH subunit [86].

Fedor *et al*. [84] recently showed that Q_10_ is an ‘optimal’ substrate for the mammalian complex I with an up to 20-fold higher *k*_cat_/*K*_m_ when compared with short-tailed quinones (Q_1_–Q_8_). In addition to ubiquinone, complex I from *T. thermophilus* can also bind menaquinone [11,87], whereas the related NDH-1 in plants is likely to employ plastoquinones [88]. It was found that the short-tailed quinones (Q_1_–Q_4_), which terminate before the interesting restriction site (see above), are more flexible in the cavity. Moreover, the increased *k*_cat_/*K*_m_ for Q_10_ could result in part from the assisted directionality of the isoprenoid tail in its motion towards the membrane. Based on the structure and size of the Q-cavity, it is also possible that more than one short-tailed quinone could fit the cavity, blocking the exit of the bound species, and thus lowering the overall turnover rate. Although the exact barrier for the Q entry/exit has not been experimentally determined, it must be faster than the overall turnover of complex I, limiting it to < 14 kcal mol^−1^. Fedor *et al*. [84] showed that the Q diffusion is not rate-limiting for complex I, and computational work [76] further indicates that Q can remain bound for microseconds. This would place the Q-dissociation process to the 10–100 µs timescales according to transition state theory (equation (7.2)), if the pre-exponential factor *κ* remains close to 1.

To probe the structure and energetics for the Q-reduction steps, Gamiz-Hernandez *et al*. [68] performed hybrid quantum/classical (QM/MM) simulations and continuum electrostatics calculations. They found that conformational change between the stacked and hydrogen-bonded binding modes of Q can modulate its redox potential and thus further also the N2 → Q electron transfer rate. Interestingly, it was also suggested that both the first and second electron transfer steps from N2 are nearly isoenergetic, placing ubiquinone at a redox potential approximately −260 mV, whereas menaquinone, which also forms similar binding poses, has a redox potential near approximately −230 mV despite its lower redox potential in membranes of approximately −80 mV. Binding properties of the latter are relevant for complex I from *T. thermophilus*, which switches to menaquinone under anaerobic conditions [11].

The low redox potential of Q in its binding site near N2, originally suggested based on electrometric measurements [89], is important for understanding the energetics of the proton-pumping machinery. As Q has a redox potential of approximately +90 mV in membranes, this implies that the motion of the Q towards the membrane is coupled to a free energy release step that could thermodynamically drive the function of the proton pump. Moreover, if menaquinone is used as a substrate, this is expected to transduce less free energy due to the lower menaquinone redox potential of approximately −80 mV in membranes.

**Scheme 1.**
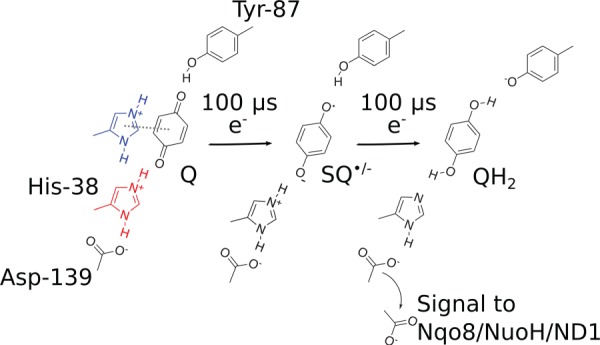
Quinone chemistry in the active site of complex I. The quinone headgroup forms a hydrogen bond with Tyr-87 of Nqo4 (*Thermus* numbering), and alternates between stacking (in blue) and hydrogen-bonded conformations with His-38 of Nqo4 (in red), which modulates the redox potential of the Q [68]. Electron transfer from NADH along the FeS chain in approximately 100 µs [61] reduces the quinone to a transient anionic semiquinone (SQ^•/−^) species. The second electron, stored at the FeS chain, e.g. at the N1a centre, is transferred in approximately 100 µs to further reduce the SQ^•/−^ species to the quinol (QH_2_) by proton transfer from His-38 and Tyr-87. This weakens the electrostatic interactions between His-38 and Asp-139, which is flipped towards the Nqo8/NuoH/ND1 subunit, and in turn triggers the pump machinery [86].

Although details of the quinone reduction chemistry still remain unclear, it is likely that electron transfer from the FeS chain leads first on a approximately 100 µs timescale to formation of anionic semiquinone, SQ^•/−^ (scheme 1). To this end, QM/MM calculations support that the semiquinone species has a p*K*_a_ lower than the surrounding Tyr-87 and His-38 residues, and thus remains anionic [68,86]. Electrometric experiments suggest that the semiquinone species is transient and does accumulate in large quantities [89]; but cf. also [90,91]. The SQ^•/−^ is likely to have a redox potential less than −300 mV [89], and the electron transfer from N2 is thus energetically disfavoured by approximately 100–150 mV (2–3 kcal mol^−1^). The second electron from NADH, stored at the FeS chain [61,72,92] will then reduce the quinone to quinol (QH_2_) by local proton transfer from Tyr-87 and/or His-38 [68,86]. This is supported by early site-directed mutagenesis experiments [79,80], which identified that these residues are central for the Q-reductase activity. QM/MM calculations [68] found that the coupled electron transfer from N2 to the SQ^•/−^ and the local proton transfer from Tyr-87/His-38 is close to isoenergetic. This implies that the local reduction and proton transfer do not provide the main driving force for the proton-pumping process, but rather the further motion of the QH_2_ species (see below).

Deprotonation of His-38 was found to weaken its hydrogen bond with Asp-139 [86], which in turn leads to flipping of the latter towards the NuoH subunit, and dissociation of the formed QH_2_ from the active site. The flipping of Asp-139 was further suggested to trigger conformational changes in the NuoH subunit, particularly at the Glu-213_H_/Glu-163_H_ site located on a flexible loop structure, which leads to a large p*K*_a_ shift, and possible proton uptake from the N-side of the membrane. The functional relevance of this ‘E-channel’ region has previously been linked to many mitochondrial diseases, and it is also supported by site-directed mutagenesis data [56–58]. Moreover, labelling studies [93–95] also qualitatively support conformational changes in these regions.

In addition to QH_2_, QH^−^ could also be a possible species that forms after double reduction of the Q. To this end, QM/MM MD simulations [68] suggest that the second proton flickers between Q and the nearby His-38. Moreover, the experimentally characterized D139N [86] and H38A ([79]; but cf. also [96]) mutants have a residual activity of approximately 5–25%, which indicates that QH^−^ could possibly also trigger the pumping process. However, due to the large desolvation penalty of QH^−^ in membranes, this species is likely to undergo protonation to QH_2_ before or upon its exit to the membrane. Such protonation steps could have interesting consequences for activation of the proton-pumping machinery.

As quinol formation is coupled with the local borrowing of protons from His-38 and Tyr-87, these sites must re-protonate before the complex I can enter the next reaction cycle. Molecular simulations indicate that the Q site is not fully sealed from water molecules [76,84], and the N-side bulk, which is approximately 12 Å away from these residues, could form contacts via such water molecules. However, although the immediate re-protonation of His-38 and Tyr-87 could help to push the QH_2_ out from its binding site near N2, this would be expected to compromise the pump, e.g. by the putative signal transmission induced by conformational changes towards the Nqo8/NuoH/ND1 subunit. It is, therefore, likely that the re-protonation is rather slow, and kinetically controlled, e.g. by conformational or protonation changes that link to the Q dynamics itself.

## 4. Proton channels in the membrane domain of complex I

The membrane domain of complex I comprises three antiporter-like subunits [49,97], NuoN/Nqo14/ND2, NuoM/Nqo13/ND4 and NuoL/Nqo12/ND5, which are homologous to multi-resistance and pH-dependent (Mrp)-type Na^+^/H^+^ transporters, for which there are currently no resolved structures. Bioinformatical studies suggest that, in particular, subunit NuoL/Nqo12/ND5 has a common ancestor with the Na^+^/H^+^ transporting MrpA [98]. It is thus also possible that these and related hydrogenases could contain a long-amphipathic HL-helix [99] that clamps the membrane domains together [100].

Each antiporter-like subunit in complex I contains two pseudo-symmetrical five-helical bundles, TM4-8 and TM9-13 [49,97]. The broken helices TM7a/b and TM12a/b (figure 2) provide additional flexibility to the bundle, similarly as in transporters [101,102].

Owing to the evolutionary link to Na^+^/H^+^ transporters, there has been a discussion, whether complex I could also pump sodium ions [103]. Indeed, some bacteria, e.g. *Vibrio cholerae,* contain a Na^+^-translocating NADH—ubiquinone oxidoreductase, Na^+^-NQR, the structure of which was recently resolved [104], but this enzyme does not have a direct evolutionary link to complex I. It has been suggested based on experiments that complex I could also have a sodium-transporting activity [105], e.g. in the deactive form of the mammalian enzyme [106]. It, nevertheless, remains unclear whether these findings have a physiological role for the function of complex I [107].

Transporters generally function by an alternative access mechanism, in which conformational changes allow for switching between *inward* and *outward* open access states [108]. Complex I has a *face*-to-*back* symmetry in which the broken TM7a/b faces the rear of the five-helical bundle with TM12a/b (related by a rotation+translation, figure 2*d*), instead of a typical five-helix inverted repeat-motif found in transporters (related by rotation + reflection). It is currently unclear whether complex I employs large-scale conformational changes to pump protons across the membrane [109], but hydration of the antiporter-like subunits leads to subtle conformational changes within the broken helix elements TM7a/b and TM12a/b [76], shown in figure 2*c*. The conformational changes at the TM7a/b link to repositioning of, e.g. Leu-214, Trp-213 and His-211, which in turn allows for hydration of the N-side input channel (figure 2*c*). In addition to the NuoN/M/L subunits, the NuoH subunit also contains a similar five-helical bundle segment, TM2-6, which is more tilted when compared with the helical bundles in the NuoN/M/L subunits.

Experiments suggest that subunits NuoH [110], NuoN [111], NuoM [112–114], NuoL [33,114,115], but also NuoK in *E. coli* [116] are important for the proton-pumping activity in complex I. Although measuring exact pumping stoichiometry is challenging, removal of subunits ND4 (NuoM/Nqo13) and ND5 (NuoL/Nqo12) causes proton pumping with approximately half of the stoichiometry of wild-type complex I [25]. It is thus possible that the NuoH, NuoN, NuoM and NuoL subunits pump one proton each. Moreover, site-directed mutagenesis experiments show that the terminal antiporter-like subunits, NuoL/Nqo12/ND5, pump protons, because mutations of conserved residues within this subunit strongly reduce the activity of complex I [33,114,115].

The antiporter-like subunits connect a chain of charged titratable residues, a conserved Lys/Glu ion pair, followed by a ‘middle’ lysine and a ‘terminal’ lysine or glutamate residues that are bridged to the middle residue by a conserved histidine (figure 2*a*). MD simulations and p*K*_a_ calculations [76] show that when the middle Lys is protonated, this induces an electric field that pulls in water molecules along the broken TM7a/b helix to the membrane interior from the N-side (figure 2*b*). To this end, bulky residues provide a restriction site that prevents the flux of water molecules across the membrane domain that could have important implications in preventing protons from leaking.

The N-side proton input channel and P-side proton output channel form by influx of water molecules at symmetry-related locations (figure 2*d*). Interestingly, these water connectivities are indirectly regulated by the conformation of the Lys/Glu ion pair within each antiporter-like subunit (figure 2*a*, *inset*). It was suggested that when the Lys/Glu ion pair is closed, the middle Lys prefers a protonated state, whereas when the ion pair dissociates by forming contacts with the neighbouring antiporter-like subunit, the p*K*_a_ of the middle Lys is lowered due to repulsion from the uncompensated Lys residue. This leads to deprotonation of the middle Lys, and closing of the contact to the N-side of the membrane.

To support the opening/closing of the Lys/Glu ion pair, it is important that the charged residues between the antiporter-like subunits form strong electrostatic interactions (see below). To this end, complex I is likely to employ an approximately 120 Å long HL-helix that clamps antiporter-like subunits together (figure 2*a*). This clamping function is indirectly supported by site-directed mutagenesis [100] and cross-linking experiments [117].

The water arrays that form within all antiporter-like subunits support efficient Grotthuss-type proton-transfer reactions both from the N-side to the centre of the antiporter-like subunits, and horizontally across the subunits from the middle Lys to the terminal Lys/Glu residue using a bridging histidine as an intermediate site. As for typical water-mediated Grotthuss-type proton transfer reactions [38,118,119], the charge rather than the proton itself accounts for the rapid charge conduction also in complex I.

## 5. Bacterial versus mammalian complex I

Recent near-atomic resolution structures of complex I from several species, including the bovine [19], ovine [18] and fungal complex I from *Yarrowia lipolytica* [17] have been resolved at an approximately 4 Å resolution. Complex I has also been resolved as part of a supercomplex that forms interactions with two complex IIIs and one complex IV [120,121]. In the supercomplex structure, the Q-exit channel from complex I faces the input channel of the Q_o_ site of complex IIIs, which could indicate substrate channelling pathways.

In addition to the structurally well-conserved core subunits, the mammalian complex I reveals the architecture of the supernumerary (or accessory) subunits that surround the core [17–19,122]. Although the exact function of these supernumerary subunits still remains unclear [21], it has been shown that they are involved in assembly of complex I (e.g. NUMM in *Yarrowia lipolytica*, [17]); in prevention of ROS formation [123]; and in the regulation of the so-called *active*-to-*deactive* (A/D) transition [124–129]. The A/D transition is a specific feature of the mammalian complex I, in which the enzyme can exist in two, catalytically distinct forms, the dormant/deactive (D) form and the active (A) form. Complex I undergoes a spontaneous transition from the A-form to the D-form, e.g., due to lack of dioxygen or in the absence of substrates particularly when the temperature is somewhat elevated. The D-form is characterized by a very slow Q-reduction process, and the A-form can be regenerated by reoxygenation in the presence of quinone. The physiological role of the A/D transition is still under debate, but it has been suggested that such activity modulation could provide a protective mechanism for complex I and possibly the complete respiratory chain under rapid change in respiratory conditions (*cf*. [126] and refs. therein). The exact molecular basis for the A/D transition remains unclear, but recent cryo-EM structures captured complex I in different conformational states, which were suggested to be related to the active and deactive forms of the enzyme [18,19]. Based on these structures, it was suggested that the A/D transition could involve a rotation around the hydrophilic domain and a bending of the membrane domain. Moreover, a loop between two helices in the ND3 subunit becomes unstructured, which could relate to the accessibility of the Q into/out from the Q-binding site [17–19]. There are also labelling studies supporting conformational changes around the ND1, ND3 and 39 kDa subunits during the A/D transition [130].

Recent analysis of global motions in complex I suggests that the bacterial and eukaryotic enzymes both comprise twisting and bending motions that structurally resemble the A/D transition captured in the recent cryo-EM structures [131]. Although these global motions are not identical in the bacterial and eukaryotic enzymes, it was found that in particular two supernumerary subunits, B13 and the 42 kDa subunits, are important for modulating these global modes [131], and could thus also be involved in regulating the A/D transition. Higher-resolution structures are, however, currently required to provide a basis for elucidating how the global motions of complex I couple to its function.

## 6. Mechanistic model for long-range proton-coupled electron transfer in complex I

It has been suggested that complex I employs direct (electrostatics), indirect (conformational) or a combination of direct and indirect coupling principles to catalyse the long-range PCET process [13,27,76,85,86,109,132–135]. Based on structural data [17–19,49], molecular simulations can be used to probe the dynamics and energetics of key intermediate steps in the PCET machinery of complex I [68,76,85,86,109,131,135,136]. The putative mechanistic model presented below is based on such detailed information that is obtained in part from computational work, but also from experiments establishing thermodynamic and kinetic boundaries for the model. Much of the computational work on the pumping mechanism in complex I in the literature so far originates from the authors’ group [68,76,77,86,109,131] or collaborators [85], with the exception of one study that reported on the involvement of the HL-helix in the pumping process [136].

The long-range PCET process in complex I is initiated by hydride transfer between NADH and FMN that leads to formation of FMNH^−^. The electrons then distribute into the FeS chain, which is coupled to dissociation of NAD^+^ from the FMN site [61]. The two electrons transfer nearly isoenergetically to the low-potential Q, bound near N2 [61,72], which reduces the quinone (Q^ox^) to quinol (QH_2_) by proton transfer from Tyr-87 and His-38 [68,86]. This reduction process is likely to also involve a conformational change from a stacked to hydrogen-bonded Q-binding mode that helps in stabilizing the reduced Q-species [68].

The QH_2_ formation leads to a charge redistribution in the Q site that induces conformational changes in the NuoH subunit that further trigger p*K*_a_ shifts in buried titratable residues, particularly in the ‘glutamate quartet’ (Glu-213/Glu-163/Glu-248 in Nqo8 and Asp-72 in Nqo7) [86], which are indirectly supported by structural and labelling studies [93,137]. MD simulations show that these residues are well connected to the N-side bulk [76,85], suggesting that the p*K*_a_ shift could be coupled to proton uptake. This is also supported by the 75% slower pumping activity in the D139N mutant [86], a residue involved in inducing conformational changes in the NuoH subunit. The proton uptake in NuoH is, however, unlikely to couple to major energy transduction events, because Q remains at a low potential in the binding site near N2 [68,89]. Instead, the energy transduction event takes place after QH_2_ is transferred towards the membrane where the Q/QH_2_ couple is at +90 mV [11,132].

To transmit the signal to the proton-pumping machinery, QH_2_ (or QH^−^; see above) could transiently bind in the Q-channel [8], e.g. near the restriction site [84], where conserved aromatic and/or cationic residues could stabilize the quinol species by dispersive and/or cation–π interactions. Binding of quinol to such a second binding site might be supported by EPR data [90,91], and qualitatively these Q-positions could also be linked to the putative quinone ‘P-state’ and ‘E-states’ of Brandt [132] that have accessibility for protonation and electron-transfer reactions, respectively, in the proposed two-state stabilization model [132].

Binding of the quinol species to this putative second high-potential binding site would bring the quinol closer to the membrane domain, which ‘pushes’ the proton in the NuoH subunit towards the NuoN subunit, e.g. to the Glu-67_K_/Glu-32_K_ site [109]. This could trigger opening of the Lys-186_N_/Glu-112_N_ ion pair in NuoN, which deprotonates the middle Lys-216_N_ by transfer of a proton towards Lys-345_N_. MD simulations [76] show that the deprotonation of the middle lysine, Lys-216_N_, leads to dehydration of the contacts to the N-side, which is expected to help in preventing the transferred proton from leaking towards the wrong side of the membrane. Moreover, model calculations suggest that the proton transfer from Lys-216_N_ to Lys-345_N_ via water molecules and His-265_N_ is favoured by opening of the central Lys-186_N_/Glu-112_N_ ion pair.

Protonation of the terminal end of the NuoN subunit leads to accumulation of charge that opens up the Lys-204_M_/Glu-123_M_ ion pair in NuoM. This lowers the p*K*_a_ of Lys-235_M_ [76], and induces proton transfer towards Glu-377_M_ via His-292_M_. In analogy to the transfer steps in NuoN, this proton transfer event is coupled to dehydration of the connectivity to the N-side. Accumulation of charge at the edge of the NuoM subunit triggers a similar step in the terminal NuoL subunit, which involves opening of the Lys-216_L_/Asp-166_L_ ion pair, deprotonation of ‘middle’ Lys-329_L_ towards Lys-385_L_ and dehydration of the connectivity from the N-side. Molecular simulations [76,109] suggest that the Asp-386_L_ forms close contacts with the P-side bulk phase, and once the proton reaches this residue, it could be easily ejected across the membrane.

This proton release step to the P-side is expected to trigger a reverse ‘backwave’: the proton release strongly increases the p*K*_a_ of the middle lysine, which, together with closing of the Lys-216_L_/Asp-166_L_ ion pair, could trigger proton uptake from the N-side to Lys-329_L_. To this end, it is possible that the conformation of the Lys/Glu ion pair also modulates the hydration of the N-side channel (see below). Closing of the Lys-216_L_/Asp-166_L_ ion pair destabilizes, in turn, the proton stored in the terminal end of the NuoM subunit (e.g. on Glu-377_M_), which is released to the P-side via the proton channel observed in MD simulations near TM12b, but not to the N-side at TM7b. This lack of connectivity to the N-side could help in providing directionality and prevent proton leaks. Release of the proton from NuoM would similarly as in NuoL lead to reclosing of the Lys-204_M_/Glu-123_M_ ion pair and re-protonation of Lys-235_M_ from the P-side. Moreover, closing the Lys-204_M_/Glu-123_M_ ion pair destabilizes the proton stored near TM12b Lys-345_N_, which is ejected to the P-side. This triggers re-protonation of Lys-216_N_ from the P-side and closing of the Lys-186_N_/Glu-112_N_ ion pair that ejects the proton, e.g. from the Glu-67/Glu-32 site to the P-side.

The steps described above lead to pumping of four protons across the membrane, and recharging of the protons from the N-side for the next cycle. To complete the pumping cycle, the QH_2_ located at the putative second binding site must exchange with an oxidized quinone (Q^ox^) species, and the proton-deficient active site Tyr-87/His-38 re-protonates from the N-side, which could take place via water molecules that are observed in MD simulations.

In the presented putative mechanism, one power stroke that results from motion of QH_2_ is coupled to conformational and electrostatic changes in NuoH subunit, and leads to both a forward and backward propagation wave across the membrane domain. This signal, propagates by direct effects that involve electrostatic coupling between residue pairs within and in between the antiporter-like subunits. Moreover, indirect coupling effects result in motion of broken helices TM7b/TM12b, hydration events and conformational changes in the conserved Lys/Glu ion pairs. The predicted backward wave needs experimental verification, but it is consistent with, e.g. the large effects caused by mutations in the NuoL subunit that perturb the Q-chemistry.

## 7. Derivation of qualitative free-energy diagrams for the long-range proton-coupled electron transfer process in complex I

Thermodynamic considerations have always played a central role for deriving molecular mechanisms in bioenergetics [9]. This can be achieved by constructing free-energy profiles for a putative molecular mechanism of interest, and test whether the model satisfies thermodynamic and kinetic boundary conditions and experimental observations. To this end, the free energies for a transition between microstates *i* and *j* are related to equilibrium constants *K_ij_* and/or redox potential differences, Δ*E*,
7.1
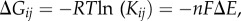
where *RT* = 0.61 kcal mol^−1^ at 310 K, *F* is Faraday's constant and *n* is the number of electrons transferred. The rates between the states *i* and *j*, *k_ij_*, are connected to the free-energy barriers (Δ*G_ij_*^‡^) by transition state theory [138],
7.2
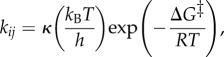
where the pre-exponential factor, *k*_B_*T*/*h* 6.4 ps^−1^. The reflection coefficient *κ* is a measure of ‘effectiveness’ of the barrier crossing, and is normally set to 1, but it can also be estimated based on Kramers' theory for diffusive barrier crossings [139].

**Figure 3.**
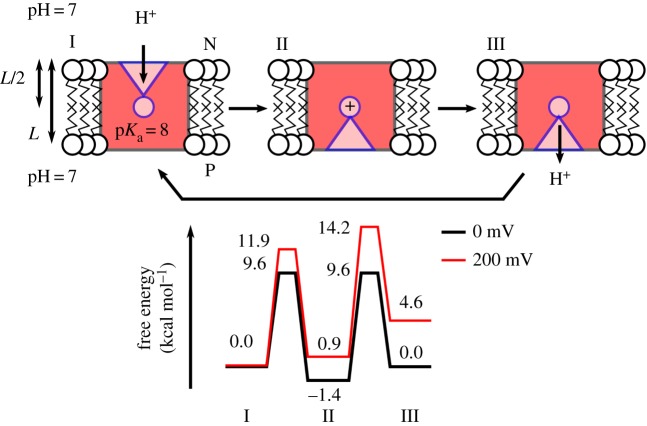
Principles for constructing free-energy diagrams for proton transfer across the membrane. The free energies are related to equilibrium constants and rates by equations (7.1)–(7.3), and the effect of a membrane potential is estimated from equation (7.4). The figure shows transfer of a proton to a single group with p*K*_a_ = 8 across the membrane from pH = 7 (N-side) to pH = 7 (P-side) without and with an external *pmf* of 200 mV. The model assumes that the proton transfer from the N-side and P-side to the buried proton loading site takes place on 1 µs timescales. The figure shows a passive proton channel, whereas a proton pump would operate with an element that modulates the p*K*_a_ of the buried site by e.g. an electron transfer site [140].

Equation (7.1) can thus be used to estimate, e.g. the energetics of transferring a charge from pH = 7 to a group with a given p*K*_a_ as follows:
7.3
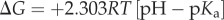
The free-energy profile for transferring a proton across a membrane with pH = 7 at both sides, using a proton pump with a single pump site with a p*K*_a_ = 8, is shown in figure 3. This example assumes that the proton uptake to the ‘middle residue’ and release take place on a 1 µs timescale.

The effect of a membrane potential, *μ*_pmf_, can be estimated by assuming that the potential drop between the N-side and the P-side is linear, leading to a perturbation of
7.4
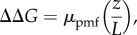
when the ion has moved a fraction of *z/L* across the membrane thickness *L*. The effect of a 200 mV *pmf* is also shown in figure 3.

Moreover, the effect of an electrostatic perturbation of a unit charge at a distance *r* can be estimated within the simplest assumption, by computing the effect of a Coulombic interaction by
7.5
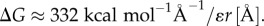


A proton pump functions by modulating the p*K*_a_ of a ‘proton loading’ site, e.g. with an electron transfer process, as in cytochrome *c* oxidase [140,141]. The pump must also secure directionality by kinetically blocking the back-leakage pathway.

Using these simple relationships described above, a qualitative free-energy profile for a putative pumping model for complex I has been derived in figure 5. In contrast with other types of PCET machinery, such as cytochrome *c* oxidase [140] or photosystem II [5], detailed data for complex I is still missing, which prevents derivation of complete free-energy diagrams.

**Figure 4.**
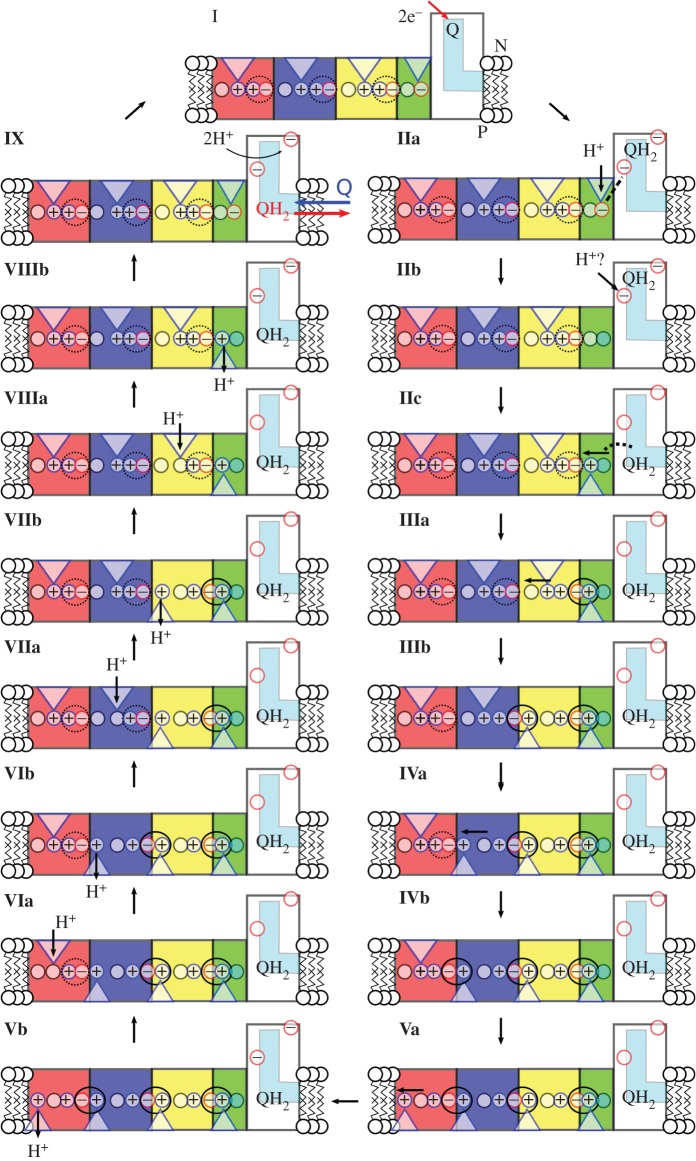
Long-range PCET model for complex I. The schematic figure shows NuoH (in green), NuoN (in yellow), NuoM (in blue), NuoL (in red), Q-channel (in cyan), water connectivity (with blue triangles), and closed and open Lys-Glu ion pairs (marked with dotted and solid black circles, respectively). Central steps: **I**: Reduction of Q by NADH. **IIa**: Formation of QH_2_ by local pT from His-38 and Tyr-87 triggers p*K*_a_ shift in the NuoH subunit. **IIb**: Proton uptake by the Glu-quartet in NuoH from the N-side. Reprotonation of His-38/Tyr-87 could also take place already in this state if the redox signal has been transmitted to the NuoH subunit (see §3). **IIc**: QH_2_ moves down in the Q-channel, which pushes the ‘NuoH’-proton towards NuoA/J/K/H. **IIIa:** Protonation of terminal residues in NuoA/J/K/H leads to accumulation of positive charge that opens up the Lys-186_N_/Glu-112_N_ ion pair. This leads to pT from Lys-186_N_ to Lys-345_N_, and dehydration of the water contact to the N-side. **IIIb:** Protonation of the terminal end of NuoN opens up the Lys-204_M_/Glu-123_M_-ion pair. **IVa:** Opening of the NuoM Lys-Glu ion pair lowers the p*K*_a_ of Lys-235_M_ that leads to pT towards Glu-377_M_, and dehydration of the water contact to the N-side. **IVb:** Protonation of the terminal end of the NuoM opens up the Lys-216_L_/Asp-166_L_ ion pair. **Va:** Opening of the NuoL Lys-Glu ion pair lowers the p*K*_a_ of Lys-329_L_ that leads to pT towards Lys-385_L_, and dehydration of the water contact to the N-side. **Vb:** Proton release to the P-side from NuoL. **VIa:** Closure of the Lys-216_L_/Asp-166_L_ ion pair and re-protonation of Lys-329_L_ from the N-side. **VIb:** Closure of the NuoL Lys-Glu ion pair destabilizes the proton at the terminal end of the NuoM subunit that is ejected to the P-side. **VIIa:** Closure of the Lys-204_M_/Glu-123_M_ ion pair and re-protonation Lys-235_M_ from the N-side. **VIIb:** Closure of the NuoM Lys-Glu ion pair destabilizes the proton stored at the terminal end of the NuoN subunit that is ejected to the P-side. **VIIIa:** Closure of the Lys-186_N_/Glu-112_N_ ion pair and re-protonation Lys-216_N_ from the N-side. **VIIIb:** Closure of the NuoN Lys-Glu ion pair destabilizes the proton stored at the terminal end of the NuoH subunit that is ejected to the P-side. **IX:** Exchange of QH_2_ with an oxidized quinone, and re-protonation of Tyr-87/His-38 from the N-side. Owing to the strong pairwise coupling between the ion-pair conformation in subunit *i* + 1 and the p*K*_a_ of the terminal residue in subunit *i*, the proton release steps are expected to propagate in the *backwave* process rather than releasing protons in subunits closer to QH_2_.

The thermodynamic properties for this model have been derived from experiments [61,72,89] and computational work [68,76,86,109], for the pumping model presented in figure 4 (see above).

In step I → IIa, the quinone is reduced to quinol in approximately 100 µs [61,72], which gives, based on equation (7.2), Δ*G*^‡^ *≈* 13 kcal mol^−1^. The rate has been estimated here based on transition state theory (equation (7.2)) rather than the Marcus theory for electron transfer (equations (2.1)–(2.2), cf. [35]). The electron transfer process is nearly isoenergetic from NADH, based on the low redox potential of Q near N2 [68,89]. This charge separation increases the p*K*_a_ of NuoH subunit residues to approximately 10 [86]. Proton uptake from the N-side (pH = 7) to titratable residues in NuoH could, therefore, be exergonic by approximately 4 kcal mol^−1^ (IIa → IIb, Δ*G* = +2.303*RT* [pH-p*K*_a_] = 1.42 × [10–7]). The rate for this proton uptake is unknown, but simulations suggest [76,109] that once the water connectivity forms on approximately 100–1000 ns timescales, the proton transfer itself might not be rate-limiting. It has, therefore, been assumed here that the proton uptake is limited by the water chain formation, which has free-energy barriers of approximately 8–10 kcal mol^−1^ (100 ns − 1 µs) based on equation (7.2).

After Q reduction and proton uptake by NuoH, the quinol moves down along its channel (IIb → IIc) that could lead to a free-energy release step because Q moves towards higher potentials. The putative transient location of Q in the tunnel has not been resolved [8], but it is assumed here that the IIb → IIc step releases approximately 60% of the free energy from the −320 mV → +90 mV motion, which would place state IIc at approximately −16 kcal mol^−1^ (equation (7.1), 60% of Δ*G* = −*nF*Δ*E* *≈* 0.6 × −19 kcal mol^−1^ = −12 kcal mol^−1^; (−4–12) kcal mol^−1^ = −16 kcal mol^−1^; *n* = 2, *F* = 96 485 C mol^−1^). Moreover, it is assumed here that the motion of Q is not rate-limiting, with a low free-energy barrier of Δ*G*^‡^ *≈* 4 kcal mol^−1^ for the IIb → IIc transition.

These initial electron transfer and Q-motion steps have charged up complex I, and the free energy is transduced in the subsequent steps into proton pumping across the membrane (IIIa → VIIIb). Opening of the Lys/Glu ion pair has a free-energy barrier of approximately 4 kcal mol^−1^ based on free-energy simulations [76], and the subsequent proton-transfer steps to the terminal residues of the antiporter-like subunits, once the ion pairs are open, are slightly exergonic (Δ*G ≈* −1 kcal mol^−1^) with Δ*G*^‡^ *<* 10 kcal mol^−1^, based on model calculations. This defines the free energies for steps IIc → IIIa, IIIa → IVa, IVa → Va, and brings the free-energy level of state Va to approximately −19 kcal mol^−1^. At this point, the terminal residues of each antiporter-like subunit have been protonated, which is followed by the stepwise release of the protons to the P-side in the subsequent steps. Both the terminal residues and the ‘middle lysines’ (see above) could have p*K*_a_s ≈ 9 [76,109], which would lead to approximately 3 kcal mol^−1^ endergonic proton release steps according to equation (7.3) (Va → Vb, VIa → VIb, VIIa → VIIb, VIIIa → VIIIb, Δ*G* = +2.303*RT* [pH − p*K*_a_] ≈ 1.42 × [9–7]). Moreover, the re-protonation of the ‘middle lysine’ from the N-side (pH = 7) would thus be exergonic by approximately 3 kcal mol^−1^ (Vb → VIa, VIb → VIIa, VIIb → VIIIa). Also, for these proton release- and uptake steps, it is assumed that the proton-transfer rate is limited by the water dynamics with free-energy barriers of approximately 8–10 kcal mol^−1^ (100 ns − 1 µs). The free-energy profiles suggest that the proton-pumping steps (Va → VIIIb) are nearly isoenergetic, which is also consistent with the reversibility of proton-pumping machinery in complex I [22,30].

The last step, VIIIb → IX, involves a re-protonation of the Tyr-87/His-38 residues at the Q site, and release of the quinol to the membrane. The re-protonation step could also take place earlier, because the Q-channel can form hydrogen-bonded contact with the N-side, and neutralization of these residues would help to push QH_2_ out from the cavity, e.g. already in IIb → IIc transition. State IX has a free energy of approximately −19 kcal mol^−1^ based on the thermodynamics boundary conditions for the cycle (NADH, −320 mV → Q, +90 mV, *n* = 2). The kinetics for step VIIIb → IX is currently unknown (dashed barrier) but the complete machinery has a turnover on millisecond timescales [12], which limits this barrier to approximately 14 kcal mol^−1^. It is interesting also to note that menaquinone has a redox potential of approximately −80 mV in membranes, which would place state IX only at approximately −11 kcal mol^−1^ if menaquinone is used as a substrate.

To derive how the free-energy profiles are affected by a membrane gradient of approximately 200 mV, all steps linked to a charge transfer across the membrane are shifted by the respective free-energy cost of such transfer steps according to equation (7.4). The complete transfer of a proton across the membrane against a 200 mV *pmf* (N-side → P-side) is endergonic by 4.6 kcal mol^−1^. The proton uptake processes to the buried ‘middle lysines’ would therefore be expected to destabilize by approximately 2.3 kcal mol^−1^ because these residues are located approximately halfway into the membrane plane. Here it is also assumed that the electric field is linear and the dielectric is constant across the membrane. This places the end state, IX, at approximately −1 kcal mol^−1^ (4 H^+^ × 4.6 kcal mol^−1^ ≈ 18 kcal mol^−1^).

It is currently not known how efficiently or at what rate complex I pumps protons across the membrane at a high *pmf*. Nevertheless, all states must be kinetically accessible, which would place boundaries on the highest free-energy barriers to approximately 20 kcal mol^−1^ (seconds timescale). The destabilization of the charge transfer steps due to the *pmf* (IIa, Vb → VIIIb) might therefore place thermodynamic boundaries on the quinol release and re-protonation steps (VIIIb → IX), as well as on the Q movement in its channel (IIb → IIc), which is here assumed to be highly exergonic. Although the Q movement is expected to provide the major power stroke in the presented pumping model, a too strong thermodynamic stabilization could lead to kinetic trapping of certain states under high *pmf*. This could thermodynamically also result in pumping with a lower stoichiometry at high *pmf*, for which there are indeed some indirect thermodynamic arguments that would favour such operation modes [27]; but cf*.* [26].

## 8. Discussion and general mechanistic implications of the proposed model

The long-range force-propagation mechanism presented here makes several predictions that can be tested experimentally and in computer simulations. Molecular simulations of transient steps along the pumping cycle provide valuable insights of residues that can be probed in site-directed mutagenesis-, labelling- or cross-linking experiments. Good candidates for mutagenesis experiments would not only be residues that block proton uptake or release from/to the P-/N-sides, but also residues that would specifically perturb the propagation energetics by stabilizing, e.g. charged intermediates along the TM7b → TM12b proton transfer steps or open/closed states of the Lys/Glu ion pair.

**Figure 5.**
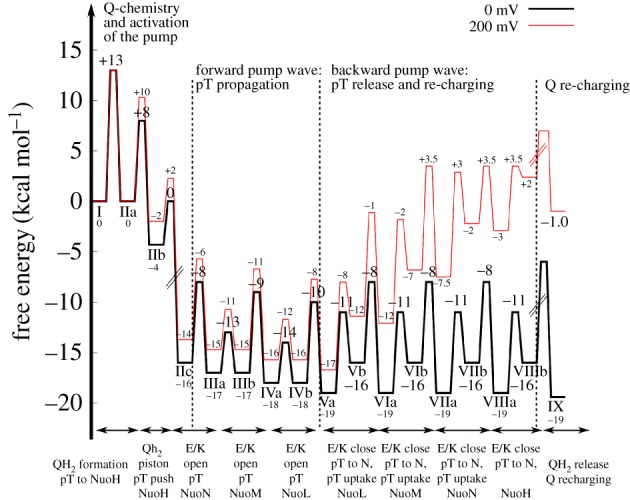
Qualitative free-energy profiles for the long-range PCET process for complex I estimated at a *pmf* = 0 mV (in black) and 200 mV (in red). Labelling of states is defined in figure 4. The p*K*_a_ of the terminal proton acceptor in the antiporter-like subunits were assumed to have a p*K*_a_ of 9–10 [76,109], and the N- and P-sides have a pH = 7. Quinone transfer along its channel was assumed to release approximately 600 mV (IIb → IIc). The thermodynamic values are estimated at *T* = 310 K and transition state energies are estimated based on transition state theory using a standard pre-exponential factor. States with dashed lines cannot be determined due to missing data. See main text (§7) for derivation of the free-energy profiles.

The pumping model described above suggest that the quinol motion from the binding site near the N2 centre towards the membrane domain could provide the main thermodynamic driving force for the pumping process (figure 5). By contrast, the signal propagation that involves conformational changes in the conserved ion pairs and horizontal proton transfer within each antiporter-like subunit is predicted to be energetically nearly degenerate. Moreover, this forward wave from H → L is only weakly affected by an external *pmf*, which supports the reversibility of the proton-pumping machinery.

When complex I functions in reverse [29], the ‘upward’ motion of the quinol from the membrane towards the N2 centre must be kinetically accessible (IIc → IIb) at high *pmf*, which places an upper bound for the exergonicity linked with quinol motion towards the membrane. A motion of a *neutral* rather than *charged* quinol species could, therefore, help to secure the reversibility of this step. The energy level of the quinol near the membrane within the NuoH subunit should also not release the full free energy stored within the Q/QH_2_ redox couple, to secure the directionality of the pump signal towards NuoL by a small driving force. On the other hand, the kinetics of the electron transfer in the reverse direction from the Q site towards NAD^+^ is not expected to have such kinetic limitations, because it is assumed to take place above the electric Stern layer of the membrane, and is thus less affected by the *pmf*. This gives a possible explanation for the unusual architecture of complex I, with the electron transfer module structurally separated from the proton-pumping membrane domain.

The putative pumping model described here also predicts that the proton release across the membrane is initiated from the terminal NuoL subunit, by ejection of the first proton across the membrane, which is further coupled to closing of the Lys/Glu ion pairs and re-protonation of the middle residues. Owing to the tight electrostatic coupling between the conformation of the ion pairs, and protonation states of the surrounding residues (middle residue/terminal residue in the previous subunits), the backwave signal probably propagates from L → M → N → H, rather than stochastically releasing the proton from subunits closer to the quinol site. This backwave is thermodynamically degenerate at 0 mV *pmf*, but becomes thermodynamically favoured in the reverse direction under 200 mV *pmf* (figure 5). The overall energetics would, nevertheless, allow to pump four protons across the membrane also at 200 mV, which would employ, with current model parameters, 95% of the free energy of the NADH/Q → NAD^+^/QH_2_ redox couple. The machinery is also predicted to become kinetically slower at high *pmf*, which could lower the pumping stoichiometry, and eventually turn the machinery in the reverse direction at high *pmf*. These features are supported by two experimental findings: (1) that complex I indeed can drive quinol oxidation and reverse electron transfer with an external pH gradient [22,29,30], and (2) that site-directed mutagenesis experiments in NuoL slow down the Q-reductase activity [32,33,53,114]. Accurate experimental determination of turnover rates and pumping stoichiometries at various *pmf*s are, therefore, important for validation of the proposed mechanism.

Although technically challenging to probe, the proposed backwave signal (figures 4 and 5) could be observed in time-resolved optical spectroscopic experiments, by measuring a time-dependent acidification pulse that is expected to form along the membrane surface and provide central boundaries for the proposed mechanistic model. This could be achieved by specific labelling of complex I with fluorescent dyes that are quenched by protons. To measure the timing of the putative NuoL, NuoM and NuoN proton-release steps across the membrane, such experimental set-ups would, however, need accurate spatial resolution (approximately 20–30 Å) but also a high time resolution (sub-microsecond).

There is an interesting thermodynamic analogy between complex I and F_1_F_o_-ATPase, which is another fully reversible enzyme of the respiratory chain. In F_1_F_o_-ATPase, proton transfer through the c-ring in the F_o_-membrane domain is employed for driving the chemical synthesis of ATP from ADP and P_i_ in the F_1_-catalytic domain—and vice versa in the hydrolysis mode [142,143]. This coupling is achieved through the rotary motion of the γ-subunit. The catalytic cycle is divided into three 120° rotary motions of the γ-subunit [144], which are in turn subdivided into 30, 65 and 25° steps in eukaryotic F_1_F_o_-ATPases [145]. Interestingly, dwell phases of this rotary motion link to ATP binding and phosphate release steps, which could, similarly as the dissociation of QH_2_ from the Q-binding site in complex I (see above), also be coupled to energy transduction in F_1_F_o_-ATPases. Rather than from the local P–O hydrolysis itself, the energy transduction could arise in part from the release/binding of the ADP and P_i_ from the active site, e.g. due to relaxation of the electrostatic repulsion between the ADP and P_i_ fragments that link with subtle structural changes [146,147]. This would indicate that the active-site structures of complex I and F_1_F_o_-ATPase, and possibly other fully reversible enzymes, are tuned in such a way that the free energies of the reactant and product states are energetically levelled, by perturbing them from their bulk reaction free energies. Such reactant/product-state levelling could favour the full reversibility of the enzymes.

The finding that the charge state of buried titratable sites regulates the conduction properties of the channel itself [76,109] seems to be a general functional feature in several energy-converting proteins. Such molecular ‘field-effect transistors', where the gating charge regulates conduction properties of the channel, might also to be employed in cytochrome *c* oxidase (C*c*O), where the reduction of the binuclear heme *a*_3_/Cu_B_ centre induces an electric field that affects water chain formation [2,148]. We have recently also observed a similar effect in the light-driven Na^+^-pump KR2, where formation of certain charged photocycle intermediates regulates the water-access and ion-conduction energetics across the two sides of the membrane [149].

An important question also arises on the maximal spatial extent of energy transduction. Could a complex I-like machinery operate on macroscopic scales or is there a physical principle limiting this to a certain length scale? The respiratory chains as a whole certainly operate on a dimension much larger than complex I. A qualitative answer probably lies in the fidelity of the elementary coupling elements that must compete with thermal energy. If each electrostatically coupled element, say 10 Å apart, would dissipate 5% of the energy (i.e. have a 95% fidelity), this would lead to a 0.2 kcal mol^−1^ loss for each interaction pair (equation (7.5), *ɛ* = 10) and would allow the signal to propagate approximately 200 Å away. Such dissipations could indeed be realistic, considering that thermal fluctuations are typically in the order of a few *k*_B_*T*s (1 *k*_B_*T* at *T* = 310 *K* = 0.616 kcal mol^−1^)*.*

## 9. Conclusion

Structural data combined with molecular simulations have allowed us for the first time to derive a molecular picture on how the respiratory complex I uses its quinone-reductase activity to pump protons up to 200 Å away from the active site. Our combined data suggest that complex I employs a combination of electrostatic changes that lead to side chain conformational flips and modulate p*K*_a_ values of titratable residues involved in the pumping machinery. These transitions take place by conformational changes in broken helices in antiporter-like membrane subunits, which further regulate the hydration state of the proton-conducting channels. Recent structural and computational data also suggest that complex I has rich global dynamics that are important for modulating the Q-dynamics and chemistry. This suggests that complex I drives its long-range PCET machinery by a combination of indirect (electrostatic) and direct (conformational) coupling principles.

## 10. Summary points of key mechanistic suggestions

— Complex I is a fully reversible redox-driven proton pump that couples quinone reduction in its hydrophilic domain to proton pumping across its membrane domain.
— Release of quinol (QH_2_ or QH^−^) from the Q-binding site could be coupled to a main energy transduction step in complex I.
— Quinol formation leads to rearrangement of ion pairs in the Nqo8/NuoH/ND1 subunit that triggers protonation changes.
— Complex I employs four putative proton channels to transfer protons across the membrane. The channels are established by water molecules at the broken helices TM7a/b and TM12a/b (and TM6 in Nqo8), and horizontally across the antiporter-like subunits.
— The proton transfer is controlled by the hydration state of the channels, which in turn is regulated by the charge state of conserved buried charged residues.
— Opening of the Glu/Lys ion pairs in each antiporter-like subunit triggers horizontal proton transfer by destabilizing the buried lysine residues in the antiporter-like subunits.
— Accumulation of positive charge at the terminal end of each antiporter-like subunit opens up the ion pair in the next subunit in the propagation of a ‘*forward*’ signal across the membrane domain.
— Deprotonation of the middle lysine residues closes contacts to the N-side, which prevents the pumped protons from leaking backwards.
— A ‘*backwave*’ signal from NuoL/Nqo12/ND5 towards NuoH/Nqo8/ND1 is proposed, in which proton uptake from the N-side and closing of the Lys/Glu ion pair releases the proton loaded in the previous neighbouring subunit.
— The pumping steps are energetically nearly degenerate, supporting the reversibility of the machinery.

## Data accessibility

This article has no additional data.
